# An Exploratory Assessment of Sleep Patterns, Burnout and Perceived Stress of a Cohort of Active Duty Military Physicians

**DOI:** 10.15694/mep.2020.000145.1

**Published:** 2020-07-10

**Authors:** Divya Ramani, Abigail Konopasky, Anthony Artino, Steven Durning, Jeffery Mikita

**Affiliations:** 1 Uniformed Services University of the Health Sciences; 2 Defense Health Agency

**Keywords:** Sleep, Actigraphy, Burnout, Stress, Military physicians

## Abstract

This article was migrated. The article was marked as recommended.

**Introduction:** Physician well-being is crucial and has the potential to impact patient safety. Many physicians across different stages of their careers experience stress, burnout, and/or decreased sleep. These factors can negatively affect physician well-being and performance and contribute to medical errors. The purpose of this study is to further understand physician well-being by examining a single cohort for patterns of sleep, burnout, and perceived stress across gender, training level, and specialty.

**Materials and methods:** A cohort of 32 practicing military physicians ranging from first-year residents to experienced attendings continuously wore an actigraphy watch for a duration of at least 5 days to capture baseline sleep patterns. On the last day of data collection, participants completed a self-reported assessment of their daytime sleepiness using the Epworth Sleepiness Scale (ESS), a two-item burnout scale adapted from the Maslach Burnout Inventory, and a 10-item perceived stress questionnaire. Data for the entire cohort were descriptively analyzed.

**Results:** The cohort had a mean sleep duration of 6.69 hours across the 5 days, with a maximum mean sleep duration of 7.90 hours, and a minimum mean sleep duration of 5.69 hours per day. Analysis stratified by gender and level of training revealed an average sleep duration of at least 6 hours across these groups. Overall, the cohort reported low perceived stress levels, low daytime sleepiness, and low burnout.

**Conclusion:** The cohort of physicians examined in the present study did not show signs of significant sleep deprivation, feelings of perceived stress, or burnout. This may be due to military culture and the structure of military training facilities that emphasize duty hour regulations. In addition, these findings may be related to the fact that military health professionals are salaried, as opposed to being on a fee-for-service schedule, and military facilities offer well-being programs.

## Introduction

The literature on physician well-being often portrays a fairly bleak story ( Raj, 2016; Dewey, Sico, and Moeller, 2019; Dimou, Eckelbarger and Riall, 2016). Physicians’ inherently challenging jobs and social and family responsibilities can all contribute to diminished sleep, increased stress, job burnout, and medical errors ( Raj, 2016; Dewey, Sico, and Moeller, 2019; Lockley *et al.*, 2007; Baldwin and Daugherty 2004). Yet these effects may not be homogenous across all physicians: prior studies reveal differences in sleep deprivation, stress, and burnout across both gender and level of training (e.g., intern, resident, attending), with women and those at lower levels of training experiencing poorer sleep and worse stress and burnout ( Raj, 2016; Dewey, Sico and Moeller, 2019; Dimou, Eckelbarger, and Riall, 2016). Hence, the purpose of this descriptive study is to specifically examine patterns of sleep, perceived stress, and job burnout together among military physicians, further exploring if there are differences across gender and training levels.

Sleep plays a vital role in ensuring optimal brain and body function ( Veasey *et al.*, 2002), but the National Sleep Foundation’s 2018 poll revealed that only 10% of Americans prioritize sleep over other aspects of a healthy lifestyle ( Owens, 2001). Sleep deprivation arises when healthy adults average less than 5 hours of sleep a day, with 5 hours of sleep nightly or two nights of total sleep deprivation over a week both causing cognitive impairment ( Veasey *et al*., 2002; Owens, 2001). Lack of sleep can lead to negative moods, anxiety, depression, irritability, confusion, and fatigue ( Durmer and Dinges, 2005). Sleep deprivation is common among professionals with demanding work schedules such as those in health care ( Weinger and Ancoli-Israel, 2002; Alhola, and Polo-Kantola, 2007). A study of internal medicine residents revealed that being on-call reduces total sleep time and negatively affects emotional equilibrium ( Rose, and Ware, 2008). In another study of 33 surgical residents, a mean sleep of 5.3 hours reduced mental effectiveness by 80%, induced a high level of fatigue, and increased the risk of medical errors ( Weinger, and Ancoli-Israel, 2002). In a national survey, first- and second-year residents who averaged 5 or fewer hours of sleep a day reported conflict with other professional staff, more accidents, the consumption of alcohol, and medication to stay awake ( Baldwin and Daugherty, 2004). The US Army Surgeon General implemented the performance triad (P3) in which sleep is considered to be one of three important components, along with nutrition and activity to maintain a healthy lifestyle in the military ( Ebrahimi and Kargar, 2018). Yet, only about 25% of Army physicians in a 2015 survey adhered to the sleep tenet i.e. adequate sleep hours ( Ebrahimi and Kargar, 2018). This study further explores sleep patterns by examining a cohort of physicians across services at a military medical hospital.

Stress, which is defined as a psychological response caused by either an internal or external stimulus, hampers mental and physical well-being. Workplace stress is a common phenomenon among physicians (Hsu *et al.,* 2018, Whitley *et al.,* 1989, Van Wietmarschen *et al*., 2018). A study of residents determined that they experienced stress a result of role ambiguity, role overload, sleep deprivation, increased workload, as well as a mismatch between job responsibilities and the individual’s knowledge and skillset in fulfilling their responsibilities (Hsu *et al.,* 2018). In a comparison among physician expertise level (intern, resident, and attending) poor sleep quality and the increased patient load was found to be a major contributor to work stress ( Whitley *et al.*, 1989). Stress levels also appear to differ by gender: female physicians reportedly experience varied and more level of stress, owing at least in part to social-career conflicts ( Dewey and Moeller, 2019; Van Wietmarschen *et al*., 2018).

This study explores the prevalence of stress in a cohort of military physicians across these identified areas of gender and level of training.

Stress is also regarded as one of the chief contributors to physician burnout. Job burnout is a syndrome characterized by depersonalization, emotional exhaustion, and a sense of low personal accomplishment ( Ishak *et al*., 2009). It occurs at a higher rate in medicine than in the general population of the United States ( Raj, 2016). A literature review revealed that burnout is prevalent across all stages of the physician’s lives: medical school, residency, and practice ( Mannam, 2019). The 2019 Medscape National Physician Burnout, Depression, and Suicide Report revealed that 44% of physicians in the United States report being burnt out, with female physicians 28% more likely than males to experience burnout ( Summers *et al*., 2019). Amongst the different specialties, internal medicine (49%), family medicine (48%), and general surgery (46%) were among the most burned-out physicians ( Kane, 2019). In a longitudinal study of resident physicians, higher rates of burnout symptoms were found among female residents and in general medicine ( Summers *et al*., 2019). Similarly, female surgeons showed higher levels of burnout and signs of depression than male surgeons ( Durning *et al*., 2014). Burnout is an issue in military physicians as well, with a recent study estimating that 26% of physician faculty in military graduate medical education experience burnout ( Dyrbye *et al*., 2018).

Conducted as a part of larger clinical reasoning study, this investigation describes the patterns of sleep, perceived stress, and burnout among a cohort of practicing military physicians in three of the specialties prone to sleep deprivation and burnout: internal medicine, family medicine, and surgery ( Dyrbye *et al*., 2018). Based on the above literature suggesting that female physicians may experience more stress and burnout than male physicians, we also explored whether these patterns differ by gender. Finally, based on concerns about sleep deprivation in residency ( West, Dyrbye, Sloan, and Shanafelt, 2009) we examine differences across levels of training (intern, resident, and attending).

## Methods

This descriptive study was conducted at Uniformed Service University of the Health Sciences (USU) and the Walter Reed National Military Medical Center (WRNMMC). Practicing physicians from primary care and surgery volunteered to participate in the study. Sleep data were collected using an actigraphy watch (Philips Respironics - Actigraphy Spectrum Plus), which participants wore for a minimum period of 5 days (for participants who wore the watch longer than 5 days, we took only the last 5 days of data). At the end of this period, as part of the larger study, participants completed the Epworth Sleepiness Scale, Perceived Stress Scale, and the two-item version of the Maslach Burnout Inventory. The data collected were analyzed using SPSS 25 and the research protocol was approved by the institutional review boards of USU and WRNMMC (MED-83-3824).

### Measurements

***Actigraphy.*** In this study, participants wore an activity-sleep monitoring watch (Philips Respironics -Actigraphy Spectrum Plus) for a minimum period of 5 days for 24 consecutive hours (except while showering, bathing, or swimming). The activity and sleep data collected were analyzed using the Actiware Software (Philips Respironics), providing information on hours of sleep per day, maximum, minimum, and average sleep time. Participants’ sleep patterns were studied as a part of their everyday routine without any manipulation. Following Veasey *et al*., 2002 we defined sleep deprivation as having average sleep of less than 5 hours per day.

***Epworth Sleepiness Scale.*** The Epworth Sleepiness Scale (ESS) is a widely used, self-report measure of daytime sleepiness ( Johns, 1991). Using a 0 (never dozing) to 3 (high chance of dozing) response scale, participants rated their recent tendency to doze/fall asleep in eight different situations. The score ranges from 0 to 24, with a score of 10 and above indicating daytime sleepiness ( Johns, 1991).

***Perceived Stress and Burnout Measures.*** To understand the well-being of this cohort, participants completed a 10-item perceived stress scale (PSS) that used a five-point response scale from 0 (never) through 4 (very often) ( Cohen, Kamarck, and Mermelstein, 1983; West, Dyrbye, Sloan, and Shanafelt, 2009). Perceived stress is the frequency with which situations in one’s life are appraised as stressful. The score ranges from 0 to 40, with scores of 13 or below indicating low stress, scores of 14 to 26 indicating moderate stress, and scores of 27 and above indicating high stress ( Cohen, Kamarck, and Mermelstein, 1983).

Participants also completed a two-item measure adapted from the Maslach Burnout Inventory (MBI), which has been shown to reliably measure physician burnout ( West, Dyrbye, Sloan, and Shanafelt, 2009; Rus & Sandu, 2013). One of the two items pertains to emotional exhaustion (“I feel burned out from my work”) and the other pertains to depersonalization (“I have become more callous towards people since I took this job”). The measure employs a seven-point Likert-type scale (from 0, “never,” to 6, “every day”). Consistent with prior reports, we considered a total score of 12 as high burnout, 6 to 11 as moderately burned out, and 5 and below as low burnout.

## Results/Analysis

A total of 32 practicing military physicians from USU and WRNMMC participated in the study; 22 were males and 10 were females (see Table 1). The sample included 16 attendings, 7 residents, and 9 interns; 26 were in primary care and 6 were in surgery (see Table 1). See Appendix A for data on all participants.

As measured by the actigraphy watch, the cohort had a mean nightly sleep of 6.69 hours ( *SD* = .66), along with mean nightly sleep we also looked at mean minimum nightly sleep of 5.7 hours ( *SD* = .97) and mean maximum nightly sleep of 7.9 hours ( *SD* = 1.16; see Table 2) over the 5-day study period. These actigraphy measures do not indicate sleep deprivation. Across gender, both male and female physicians obtained more than 6 hours of sleep, with females getting slightly more sleep than males ( Table 2). Finally, physicians at all levels of training appeared to obtain adequate sleep, with interns sleeping slightly more on average than residents who slept slightly more on average than attendings (see Table 3). Overall, only 15.6% ( *n* = 5) of participants had mean sleep amounts of less than 6 hours: two attendings, two residents, and an intern, all male (see Table 3).

Similarly, physicians’ self-reports on the ESS revealed little daytime sleepiness on average ( *M* = 6.81, *SD* = 3.89). Across gender, 23% of males and 10% of females reported daytime sleepiness (see Table 3). While standard deviations were high, residents reported slightly higher daytime sleepiness than interns and attendings (mean of 8.7 on ESS for the former versus 6.6 and 6.1 for the latter; see Table 2).

Results from the PSS indicated that physicians generally experienced low stress, with a mean of 10.6 ( *SD* = 6.3). However, gender appeared to be a factor, with only about 32% of males reporting moderate to high stress, compared to 60% of females (See Table 3). Across the level of training, there was little difference, with 33.3 % of interns, 33% of residents, and 44% of attendings reporting moderate to high stress (See Table 3).

Finally, this cohort reported low levels of burnout overall, with a cohort mean of 3.66 ( *SD* = 2.91) on the MBI. However, burnout, like stress, appeared to differ according to gender, with 40% of females and only 27% of males reporting moderate to high burnout (see Table 3). Notably, only 11% ( *N* = 1) of interns reported moderate to high burnout, compared to 57% of residents and 31% of attendings.

**Table 1.** Demographic information

	Intern	Resident	Attending
**Gender**			
Male	4	5	13
Female	5	2	3
**Specialty**			
Primary care	6	6	14
Surgery	3	1	2

**Table 2.** Comparison of study variable means across gender and level of training

	Minimum sleep *M* ( *SD*)	Maximum sleep *M* ( *SD*)	Average sleep *M* ( *SD*)	Perceived sleepiness *M* ( *SD*)	Perceived Stress *M* ( *SD*)	Burnout *M* ( *SD*)
**Gender**						
Male	5.5 (.85)	7.5 (1.03)	6.5 (.60)	7.2 (4.39)	9.6 (6.6)	3.36 (3.06)
Female	6.1 (1.14)	8.6 (1.17)	7.1 (.61)	5.9 (2.42)	12.5 (5.31)	4.3 (2.58)
**Level of Training**						
Intern	6.7 (4.27)	8.5 (1.3)	6.9 (.66)	6.6 (4.27)	8.7 (6.43)	2.4 (1.74)
Resident	5.8 (1.28)	7.6 (.82)	6.7 (.85)	8.7 (5.18)	12 (2.76)	5.4 (2.44)
Attending	5.5 (.79)	7.6 (1.09)	6.5 (.57)	6.1 (2.93)	10.9 (7.33)	3.6 (3.34)

**Table 3.** Percentage of participants experiencing sleep deprivation, daytime sleepiness, stress, and burnout

	Gender		Training Level		
	Male *N* =22	Female *N* =10	Intern *N* = 9	Resident *N* = 7	Attending *N* =16
Sleep deprivation (˂ 5 hours for 2 nights)	5% ( *N* = 1)	0	0	0	6% ( *N* = 1)
Daytime sleepiness (≥ 10 on ESS)	23% ( *N* = 5)	10% ( *N* = 1)	22% ( *N* = 2)	29% ( *N* = 2)	13% ( *N* = 2)
Moderate to high stress (≥ 14 on PSS)	32% ( *N* = 7)	60% ( *N* = 6)	33% ( *N* = 3)	43% ( *N* = 3)	44% ( *N* = 7)
Moderate to high burnout (≥ 6 on MBI)	27% ( *N* = 6)	40% ( *N* = 4)	11% ( *N* = 1)	57% ( *N* = 4)	31% ( *N* = 5)

## Discussion

This study examined three components of well-being - sleep, stress, and burnout - and their patterns across gender and level of training in a cohort of 32 military physicians. Overall, this cohort appeared to get an adequate amount of sleep and indicated little burnout and stress, with some small, but not statistically significant, differences emerging across gender and levels of training. These results are encouraging and could be the result of a number of factors. In particular, the participants in this study worked in an academic military medical facility that strictly follows duty hour regulations and, as part of the armed services, is very much “mission-focused.” That is, military physicians have been shown to report higher job satisfaction, as well as a sense of being a part of something “bigger” than other physicians ( Rus and Sandu, 2013). Moreover, these physicians are salaried and are not required to see a specified number of patients per day, as some civilian facilities require; a fact that perhaps functions to reduce overall stress and burnout.

Another factor that may have contributed to these results is that participants were allow to select their study times, and tended to do so during times of reduced workload (e.g., during a “light” rotation). As such, these reduced workload periods may have allowed participants to get more sleep and perhaps feel less stressed and burned out than would be typical. Notwithstanding the uniqueness of the cohort studied here, this work provides a window into the *possibilities* that can be achieved when the appropriate work conditions are in place. These findings are both in line with the National Sleep Foundation recommendations, and they are an improvement over the 2015 survey of U.S. Army physicians ( Kassam *et al.,* 2015; McCormick *et al*., 2012).

It is important to note, however, that even in this small cohort of physicians, there were sub-groups who appeared more likely to fall below the recommended sleep amounts and/or who experienced stress and burnout. While female physicians in this cohort got more hours of sleep than males (contrary to the finding of other studies ( Rus & Sandu, 2013) more of these women reported being moderately to highly stressed and burned out, which seems to align with the literature ( Dewey, Sico, and Moeller, 2019; Ebrahimi and Karger, 2018; Summers *et al*., 2019; Dyrbye *et al.,* 2018). Moreover, the results by level of training indicate that, for this cohort, interns and residents got more sleep than attendings. This may be due, in part, to the fact that interns and residents were often given time off from clinic or the wards to participate in this study. Alternatively, it could have been the result of attendings have no duty hour regulations. Further research should explore these trends. Meanwhile, interns and attendings (compared to residents) reported higher levels of stress while residents (compared to interns and attendings) reported higher levels of burnout. Residents may have expressed higher levels of burnout due to working close to duty hour regulations. However, this is a hypothesis and should be further tested in larger studies.

This study has several important limitations. First, it was a single-institution study that examined a relatively small number of participants. Also, participants were likely to be on less time-consuming rotations that would be typical among trainees and attending physicians. That said, a strength of this study was the study design, which required participants, of multiple specialties and levels of training, to wear the actigraphy watches continuously over a five-day period.

## Conclusion

The importance of physicians is well established. This study examined several different components of well-being and their patterns across a cohort of 32 military physicians. In contrast to prior work conducted nationally, findings from this study revealed a near-optimal level of sleep, as well as low levels of stress and burnout across participants. There were, however, some small, but not statistically significant, differences in sleep and well-being across gender and level of training. While it is reassuring that this cohort of physicians appeared to be well-rested, unstressed, and not burned out, there is a need to further examine these components in larger investigations across more diverse provider populations.

## Take Home Messages

• The cohort of 32 military physicians report an optimal level of sleep along with low stress and burnout.
• It is essential to examine factors leading to optimal sleep and low stress and burnout across a diverse population.
• To examine and find ways of implementing a working system promoting physician well-being.

## Notes On Contributors

**Divya Ramani**, MS, Research Associate, Health Profession Education, Department of Medicine, Uniformed Services University of Health Science.

**Abigail Konopasky**, PhD, Assistant Professor of Medicine, Health Profession Education, Department of Medicine, Uniformed Services University of Health Science.

**Steven J Durning**, MD, PhD, Professor of Medicine and Pathology, Director, Graduate Programs in Health Professions Education, Uniformed Services University of Health Science.

**Anthony R. Artino**, Jr., PhD, CAPT, MSC, USN, Professor and Deputy Director, Health Professions Education, Uniformed Services University of Health Science.

**Jeffery Mikita**, MD, FACP, FCCP, CHSE, COL, MC, USA, Division Chief, Defense Health Agency.

## Appendices

### Appendix A: Sleep, stress, and burnout scores across all participants

**Table:**

ID #	Training level	Gender	Min. sleep	Max. Sleep	Avg. Sleep	Epworth score	Stress score	Burnout score
1	Attending	Male	4.72	6.84	5.68	4	4.00	1
2	Resident	Male	5.07	6.28	5.69	5	16.00	3
3	Intern	Male	4.75	6.44	5.74	4	2.00	1
4	Attending	Male	3.63	7.23	5.77	4	10.00	1
5	Resident	Male	3.98	6.98	5.86	7	9.00	6
6	Attending	Male	5.55	6.28	6.00	4	3.00	1
7	Attending	Male	5.38	7.28	6.12	8	16.00	6
8	Attending	Male	5.31	6.88	6.12	7	20.00	2
9	Attending	Male	5.67	6.41	6.19	9	7.00	0
10	Attending	Male	5.38	8.23	6.23	5	27.00	10
11	Attending	Female	5.15	7.47	6.25	5	17.00	2
12	Intern	Male	4.78	7.75	6.41	16	7.00	1
13	Resident	Male	5.37	7.93	6.48	15	9.00	6
14	Attending	Male	5.20	7.97	6.52	12	7.00	4
15	Intern	Female	3.96	10.31	6.53	8	9.00	1
16	Attending	Male	5.84	7.22	6.62	5	3.00	0
17	Intern	Female	5.10	7.85	6.63	6	3.00	2
18	Attending	Female	6.17	7.46	6.84	5	18.00	7
19	Attending	Male	6.70	8.12	6.91	3	3.00	1
20	Resident	Female	5.97	8.60	6.96	5	10.00	3
21	Resident	Male	5.96	7.40	6.99	17	14.00	4
22	Attending	Female	6.22	7.79	7.07	9	14.00	9
23	Intern	Male	6.48	8.04	7.11	6	2.00	1
24	Resident	Male	6.80	8.05	7.13	8	12.00	10
25	Attending	Male	5.18	11.03	7.19	4	3.00	2
26	Intern	Male	6.06	8.25	7.31	3	16.00	3
27	Intern	Female	6.79	7.91	7.35	2	6.00	4
28	Attending	Male	6.63	8.29	7.45	2	14.00	8
29	Attending	Male	6.68	8.10	7.50	11	9.00	3
30	Intern	Female	6.77	10.61	7.56	10	15.00	3
31	Intern	Female	6.89	9.64	7.850	5	19.00	6
32	Resident	Female	8.02	8.41	8.20	4	14.00	6
