# Supplementary material for: An Exploratory Assessment of Sleep Patterns, Burnout and Perceived Stress of a Cohort of Active Duty Military Physicians
Source: MedEdPublish (2016). 2020 Jul 10;9:145. [Version 1] doi: 10.15694/mep.2020.000145.1 (PMC10702675; doi:10.15694/mep.2020.000145.1)
Supplement: Peer review history

# Reviewer report: Reviewer response for version 1

**DOI:** 10.21956/mep.20017.r30533

**Published:** 2021-02-10

**Authors:** Richard Hays

**Affiliations:** 1 James Cook University

This review has been migrated. The reviewer awarded 4 stars out of 5 I also think that this is an interesting contribution. In a sense it is a 'negative' finding - using the selected measures, military physicians working in their normal, non-combat zone positions seem not to have significant sleep disturbances or stress. That is good news for the patients in their care and senior managers. It may show that rosters and support are appropriate within those hospitals. There are of course other contexts in which military physicians work so it may be useful to repeat the measures, not just with other cohorts, but also in those other contexts to explore the impact of context on sleep and stress.

Reviewer Expertise:

NA

No decision status is available

# Reviewer report: Reviewer response for version 1

**DOI:** 10.21956/mep.20017.r30530

**Published:** 2020-10-31

**Authors:** Ken Masters

**Affiliations:** 1 Sultan Qaboos University

This review has been migrated. The reviewer awarded 3 stars out of 5 An interesting paper on sleep patterns, burnout and perceived stress of active duty military physicians. The context of the need for good sleep is well established in the Introduction, and the study has been well-conducted. The sample is small and volunteers only, but the results are still interesting.Some issues that I would like to see addressed:• It would be useful if the authors could give a brief context about the setting (more than simply the name). This is partially because, in many places around the world, the term “Active Duty” in the military usually refers to being in a combat zone, and this does not appear to apply in this case. (The reader can try to get a little more from the Discussion, but there are snippets only).• In the results, several comparisons of the results are made (e.g. females had more sleep than males), but there is no indication of statistical significance. Could this please be determined and mentioned? (This is alluded to briefly in the Discussion, but should come into the Results first, and, sometimes the Discussion appears to discuss the differences as if they are statistically significant, while, early in the Discussion, it is stated that they are not).• Although we are told the size of the sample, it would be useful to know the total complement at the institutions (even if just approximately) and also the respective totals of the various sub-groups; this will allow for a deeper insight into the representativity of the sample (For example, the large difference between Male and Female in the “Attending” – is this representative?). The other sub-groups also would benefit from a comparison. This is especially important because of the small sample size.I look forward to Version 2 of the paper in which these issues addressed.

Reviewer Expertise:

NA

No decision status is available

# Reviewer report: Reviewer response for version 1

**DOI:** 10.21956/mep.20017.r30531

**Published:** 2020-07-14

**Authors:** Barbara Macpherson

**Affiliations:** 1 Leeds Institute of Medical Education

This review has been migrated. The reviewer awarded 4 stars out of 5 An interesting study that has further utility. It would be beneficial to explore the contributory factors to the noted gender differences in perceived stress and burnout.

Reviewer Expertise:

NA

No decision status is available

# Reviewer report: Reviewer response for version 1

**DOI:** 10.21956/mep.20017.r30532

**Published:** 2020-07-10

**Authors:** Felix Silwimba

**Affiliations:** 1 University of Lusaka

This review has been migrated. The reviewer awarded 5 stars out of 5 this is a good study on sleep deprivation and stress among salaried physicians. the findings are informative. the study can be replicated in developing countries where physicians are salaried but complain about low pay .

Reviewer Expertise:

NA

No decision status is available
